# Deep Neural Network Analysis of Pathology Images With Integrated Molecular Data for Enhanced Glioma Classification and Grading

**DOI:** 10.3389/fonc.2021.668694

**Published:** 2021-07-01

**Authors:** Linmin Pei, Karra A. Jones, Zeina A. Shboul, James Y. Chen, Khan M. Iftekharuddin

**Affiliations:** ^1^ Vision Lab, Department of Electrical & Computer Engineering, Old Dominion University, Norfolk, VA, United States; ^2^ Department of Pathology, University of Iowa Hospitals & Clinics, Iowa City, IA, United States; ^3^ Department of Radiology, Division of Neuroradiology, San Diego VA Medical Center, La Jolla, CA, United States; ^4^ Department of Radiology, Division of Neuroradiology, UC San Diego Health System, San Diego, CA, United States

**Keywords:** brain tumor classification and grading, glioma, central nervous system tumor, radiomics, molecular, deep neural network, cellularity, *IDH* mutation

## Abstract

Gliomas are primary brain tumors that originate from glial cells. Classification and grading of these tumors is critical to prognosis and treatment planning. The current criteria for glioma classification in central nervous system (CNS) was introduced by World Health Organization (WHO) in 2016. This criteria for glioma classification requires the integration of histology with genomics. In 2017, the Consortium to Inform Molecular and Practical Approaches to CNS Tumor Taxonomy (cIMPACT-NOW) was established to provide up-to-date recommendations for CNS tumor classification, which in turn the WHO is expected to adopt in its upcoming edition. In this work, we propose a novel glioma analytical method that, for the first time in the literature, integrates a cellularity feature derived from the digital analysis of brain histopathology images integrated with molecular features following the latest WHO criteria. We first propose a novel over-segmentation strategy for region-of-interest (ROI) selection in large histopathology whole slide images (WSIs). A Deep Neural Network (DNN)-based classification method then fuses molecular features with cellularity features to improve tumor classification performance. We evaluate the proposed method with 549 patient cases from The Cancer Genome Atlas (TCGA) dataset for evaluation. The cross validated classification accuracies are 93.81% for lower-grade glioma (LGG) and high-grade glioma (HGG) using a regular DNN, and 73.95% for LGG II and LGG III using a residual neural network (ResNet) DNN, respectively. Our experiments suggest that the type of deep learning has a significant impact on tumor subtype discrimination between LGG II *vs*. LGG III. These results outperform state-of-the-art methods in classifying LGG II *vs*. LGG III and offer competitive performance in distinguishing LGG *vs*. HGG in the literature. In addition, we also investigate molecular subtype classification using pathology images and cellularity information. Finally, for the first time in literature this work shows promise for cellularity quantification to predict brain tumor grading for LGGs with *IDH* mutations.

## Introduction

Gliomas are primary brain tumors that originate from glial cells. Survival in patients with gliomas is dependent on the tumor type and grade. According to a recent report, five-year survival is 94.1% for pilocytic astrocytoma [lower-grade glioma (LGG) grade I] yet it is only 5.6% for glioblastoma [high-grade glioma (HGG) grade IV] (1). Overall, 94.1% of patients with pilocytic astrocytoma, 57.6% of patients with anaplastic oligodendroglioma (LGG grade III), and 30% of patients with anaplastic astrocytoma (LGG grade III) survived five years after diagnosis (1). Therefore, accurate tumor classification and grading is required for proper treatment planning and assessing overall prognosis in clinical practice. The classification and grading of gliomas has evolved over time, and modern classification of gliomas was first published by the World Health Organization (WHO) in 1979 (2).

Prior to 2016, the WHO standard for tumor classification and grading of central nervous system (CNS) tumors was entirely based on histologic appearance. CNS tumors are classified according to the microscopic similarities with different putative cells of origin and differentiation levels (3). For grading of diffuse gliomas, the histological features of mitotic activity, microvascular proliferation and necrosis are used. There are many studies in the literature for tumor grading using histopathology images (4–6). With the publication of the updated CNS WHO in 2016, it was determined that histopathology for tumor classification and grading was no longer accurate in isolation (3). Therefore, molecular data combined with histology has become the new standard for CNS tumor classification (7–10). With regards to diffuse gliomas, isocitrate dehydrogenase (*IDH*) mutations have been identified as a major criterion (7).

Recently, due to the rapid progress in molecular insights into CNS tumors, the Consortium to Inform Molecular and Practical Approaches to CNS Tumor Taxonomy (cIMPACT-NOW) has been established to provide practical recommendations for CNS tumor classification (11). The cIMPACT-NOW has been updating the current WHO criteria to precisely determine tumor type and subtype using both histology and genetic information (12–14).

To accurately classify glioma grade following the current WHO CNS tumor classification criteria, both histology and genetic information are required. In addition, advancement in deep neural networks (DNN) has enabled rapid progress in many fields (5, 15–17). DNN analysis is capable of automatically learning features from raw data and thus alleviates the need for a handcrafted feature. Convolutional neural network (CNN) is a typical DNN with a convolutional layer. Both CNN and DNN structures have been extensively used in the integration of pathology images and molecular information. The accurate glioma classification and grading may provide personalized treatment for patients with brain tumor.

This work proposes a joint analysis of histopathology with integrated molecular data using DNN for brain tumor type and grading following the 2016 WHO criteria. The work utilizes digital pathology images and four key molecular features (*IDH1/2*, 1p/19q, *ATRX*, and *MGMT*) to obtain improved tumor classification and stratification accuracy. In addition, a specific shape-based measure for abnormal cell nuclei known as cellularity (18) is investigated for its efficacy in tumor classification. Cellularity is used to indicate the probability of cancerous cells from the whole slide images (WSI). Specifically, our work discovers the potential role of cellularity in tumor histopathology image and *IDH1/2* mutation status for grading stratification within lower grade (grade II and III) gliomas.

The remaining sections are organized as follows: Section III introduces the proposed method, including image pre-processing and convolutional neural networks. Section IV describes the data materials. Section V discusses the experiment. Conclusions are in Section V.

## Background and Related Work

CNS tumor classification and grading has been an intense research area. Based on the different types of patient data, the latest research on tumor classification and grading is generally categorized in three groups: digital pathology-based, structural MRI-based, and proteomics/genomics-based. The following subsections briefly review tumor classification using histopathology, MRI and proteomics data.

### Digital Pathology-Based Method

The histologic appearance of tumors has been the primary source for glioma classification and grading prior to the most recent WHO based on features such as nuclear atypia, mitotic activity, microvascular proliferation and necrosis. As of the recently updated WHO glioma classification criteria, pathology is still one of the sources for CNS glioma classification with integration of genetic data. There is a new trend towards using digital pathology images, particularly whole-slide imaging, to assist with classification separate from classic microscopic examination. Nuclei and tissue segmentation on haematoxylin & eosin (H&E) stained pathology digital images is a common method for this analysis. Kong et al. proposed a computer-aided classification method for grading of neuroblastic differentiation on whole-slide imaging (WSI) histology images (19). By using a method called sequential floating forward selection (SFFS), the authors first segment nuclei, extract hand-crafted features, apply feature selection method and finally use k-nearest neighbor for classification. Barker et al. proposed an automated brain tumor type classification in whole-slide digital pathology images using local representative tiles (6). In another work, nuclei segmentation is obtained by using hysteresis thresholding and watershedding, feature selection, and an Elastic Net Classification for brain tumor grading. In (5), Mousavi et al. proposed automated brain tumor grade discrimination based on spatial domain analysis. The authors developed a method for cell segmentation and a customized operation of spatial and morphological filters to identify microvascular proliferation, then applied a hierarchical decision for LGG and HGG classification. Reza et al. proposed a computational cell nuclei morphologic feature analysis technique to characterize gliomas in digital pathology images (20). Wang et al. used a support vector machine (SVM) network for glioma grading in digital pathology images. Yonekura et al. proposed an improved disease stage classification with a convolutional neural network for glioma histopathology images (21). They obtain classification accuracy of 87.15% for differentiating LGG and HGG. Ertosun et al. proposed a glioma grading method using convolutional neural networks (CNN) (16) and mitosis analysis for glioma classification. In (22), the authors proposed morphologic features, including mitosis and apoptosis, to improve glioma classification using a CNN. Even though histopathology-based tumor grade classification has been the standard of care, there can be high intra- or inter-observer variability (4, 23). Because of this variability in tumor grade classification using only tumor morphology, the updated WHO integrated genetic information to better classify gliomas and help guide clinical decision-making for treatment planning and management of tumor patients (7, 10, 24, 25).

### Structural MRI-Based Method

Standard clinical practice of biopsy or resection and then pathologic assessment for brain tumor classification is invasive and, therefore, non-invasive structural MRI may be an alternative source for glioma type and subtype classification (26). There are many works using MRIs for glioma classification in the literature. One of disadvantages of these conventional imaging methods is the need to extract hand-crafted features before further analysis. To overcome the issue, deep learning-based methods are proposed for glioma grading on MRI images. Sajjad et al. proposed a brain tumor classification using deep CNN with extensive data augmentation (27). Ye et al. propose a glioma grading based on 3D multimodal CNN and privileged learning (28). Deepak utilized a deep CNN features *via* transfer learning for brain tumor classification (29). The current WHO criteria for glioma grading requires both genomics and phenomics information. Only MRI-based glioma grading may be a complementary approach and may not be suitable for clinical use (30–33).

### Molecular-Based Method

Molecular studies of brain tumors have been critical to understanding the genetic underpinnings of neoplasms. For infiltrating gliomas, molecular classification more reliably reflects underlying tumor biology than traditional morphology (34). Molecular underpinnings of primary CNS tumors have changed the process of tumor diagnosis and classification (34). *IDH1* or *IDH2* mutations have been shown to be present in about 80% of grade II and grade III LGG and previously designated “secondary glioblastomas” (HGG) (35). Patients with *IDH*-mutated gliomas have significantly longer survival than for those with *IDH* wild-type tumors (9). Molecular alterations, such as *IDH 1/2* mutations*, ATRX* mutations, 1p/19q codeletion*, TERT* promoter mutations, and *MGMT* promoter methylation have also been highly studied for glioma molecular classification and prognostication (36).

Following the relationship between *IDH* mutation status and glioma classification, Chang et al. utilized a residual convolutional neural network to determine *IDH* status in low- and high-grade glioma from MR imaging (37). By analyzing Japanese glioma patients with *IDH* mutations, Mukasa et al. found that *IDH* mutations with intact chromosomes 1p/19q is useful when assessing prognosis of LGG grade III patients (38). This finding was further confirmed by analysis of The Cancer Genome Atlas Research Network (39). 1p/19q co-deletion is a biomarker of oligodendrogliomas and predicts better survival for both grade II and grade III oligodendrogliomas (40). The presence of 1p/19q co-deletion has a role as an important positive prognostic biomarker of disease. In addition, in infiltrating astrocytic neoplasms, a strong association has been found between *IDH* canonical mutations and alpha thalassemia/mental retardation syndrome X-linked (*ATRX)* gene mutations, whereas 1p/19q codeletion and *ATRX* mutations barely exist simultaneously (41). In combination with *IDH* mutations, *ATRX* mutation status is one of the critical defining markers used for molecular classification of gliomas. Among infiltrating grade II and grade III astrocytomas, 75% show *ATRX* gene mutations (42). Leeper et al. proposed an improved molecular classification method using 1p/19q codeletion, *IDH* mutations, and *ATRX* mutations for grade II diffuse gliomas (43). Promoter meythlation of the O6-methylguanine-DNA methyltransferase (*MGMT*) and *IDH1/IDH2* has a particularly high prevalence in LGG (44), and methylation of the *MGMT* promoter is predictive for treatment response in glioblastoma patients (45). In Reference (25) the authors study glioma groups based on 1p/19q status and *IDH* and telomerase reverse transcriptase (*TERT*) promoter mutations in tumors. They found that molecular groups are interdependently associated with overall survival among LGG grade II and grade III patients, but not among patients with glioblastoma.

Even though these works achieve good performance on glioma classification, there are still drawbacks. According to the WHO, using only histologic examination may not be adequate for robust low-grade glioma (LGG) classification, in particular when deciding between an astrocytoma and oligodendroglioma in which there can be a lot of overlap (3). Conventional MRI can be used as an alternative source, but it is not the standard source for glioma grading and not always 100% accurate. Therefore, we propose enhanced tumor classification using deep convolutional neural network (DNN) analysis following the 2016 WHO guidelines integrating phenotypic and genotypic information. In addition, we also experiment with the efficacy of a morphologic cellularity feature to augment glioma type and subtype grade classification.

## Method

In the section, we discuss the proposed method using DNN for glioma type and subtype grading. The subsections include region-of-interest (ROI) extraction, image pre-processing for color normalization and cellularity computation.

### ROI Selection

Considering the massive size of a whole slide image (WSI), which may be larger than 1 GB, extraction of ROI is desired. In the literature, splitting of the WSI into tiles and then using one or more tile(s) as the ROI has been reported (6, 16, 46). However, we argue that the tiles may not be representative.

To effectively select ROIs from a WSI, we propose a new strategy that utilizes an over-segmentation technique. Instead of splitting WSI into tiles, we apply an over-segmentation technique to select the ROI as shown in **Supplementary Figure 1**. We obtain thumbnail images of the WSI, then perform over-segmentation (47) on the thumbnailed image to produce many super-pixels based on the tissue similarity. Subsequently, we sort the super-pixels according to the mean intensity and select top candidate super-pixels with low mean intensity at 10-percentile of all super-pixels, which reflects cell proliferation and cellular density. Using centroid as the center of the selected super-pixel, we compute the relative location in the WSI. Finally, we crop the image with desired size as the final object from the WSI using the relative location.

An appropriate example of selecting ROI from WSI using over-segmentation is shown in **Supplementary Figure 2A**. Note that using a simple pen-marker on WSI may result in a wrong ROI selection as shown in **Supplementary Figure 2B**. Hence, human intervention may be needed for cases such as shown in this example.

### Color Normalization of WSI

In pathology, tissue sample images are stained with a combination of hematoxylin and eosin (H&E). Hematoxylin binds to nuclei with a bluish-purple color, and eosin stains acidophilic proteins with a red-pink color. The stained tissue can be digitally imaged and are easy to share and analyze with computer algorithms (48). Color normalization can help both pathologists and software in comparing different tissue samples by standardizing the image appearance. In this work, we utilize a structure-preserving color normalization and sparse stain separation proposed in (49) to normalize H&E stained tissue images. A given RGB image is converted to an optical density (*X*) based on Beer-Lambert law, then the stain separation is decomposed by non-negative constraints on the stain density (*L*) and color appearance matrix (*W*), which yields,

(1) $$\underset{W,L}{\min}\frac{1}{2}{\left\|X-WL\right\|}_{F}^{2}+\lambda \sum_{j=1}^{r}{\left\|L(j,:)\right\|}_{1},$$

where *j* is the stain index. Then, the stain separation of source (*X_s_*) and target *X_t_* images are factorized into color appearance and stain density maps (*W_s_L_s_* and *W_t_L_t_*). To preserve structure color normalization, we normalize the color appearance of a source image *s* to of a target image *t*. Finally, the color normalized image of the target image is computed as:

(2) $$L_{s}^{norm}\left(j,:\right)=\frac{L_{s}\left(j,:\right)}{L_{s}^{RM}\left(j,:\right)}L_{t}^{RM}\left(j,:\right),\;j=1,\cdots ,r,$$

(3) $$X_{s}^{norm}=W_{t}L_{s}^{norm},$$

where $L_{i}^{RM}=RM\left(L_{i}\right)\in R^{r\times 1},\;i\in s,t$ and RM computes the pseudo maximum of each row vector at 99%. The registered normalized source image is represented by:

(4) $$I_{s}^{norm}=I_{0}exp\left(-X_{s}^{norm}\right),\;$$

where *I*_0_ is the illuminating light intensity on the sample (49).

**Supplementary Figure 3** shows three examples of color normalization for different types of H&E tiles. **Supplementary Figure 3A** shows LGG grade II oligodendroglioma with mutant *IDH*, wild-type (WT) *ATRX*, 1p/19q codeletion and methylated *MGMT*. **Supplementary Figure 3B** is LGG grade III astrocytoma with mutant *IDH*, mutant *ATRX*, intact 1p/19q, and unmethylated *MGMT*. Finally, **Supplementary Figure 3C** shows HGG glioblastoma with WT *IDH*, WT *ATRX*, intact 1p/19q, and unmethylated *MGMT*.

### Cellularity Computation in WSI

Assessment of cellularity is an important component of tumor burden assessment. Cellularity is usually estimated by pathologists in clinical practice and has been used in breast cancer analysis (18, 50). The cellularity of a given image is computed as the ratio of the area of a cancerous cell over the whole image area. To identify the cancerous cell in computer-aided methods, nuclei segmentation is desired. There are many works on nuclei segmentation, including conventional machine learning-based and advanced deep learning-based methods (19, 51, 52). In general, LGG grade II is defined to have a lower cellularity value while HGG has a higher value of cellularity. In some work, cellularity is suggested to be calculated as the ratio of dilated cancerous cell over the whole image area. Both Akbar, and Peikari et al. applied image dilation, and then computed the cellularity (18, 53). In our work, we investigate efficacy of cellularity in brain tumor WSI with different image dilation size for glioma grading.

### Proposed Tumor Grading Method

We use a cascaded convolutional neural network (11) as the underlying model for tumor grading. A multi-class (LGG grade II, LGG grade III, and HGG grade IV) classification problem is posed as a stepwise binary classification problem. In the first step, we discriminate HGG and LGG using regular DNN. For LGG, we further apply a residual neural network [ResNet (54)] to distinguish between LGG II and III. The proposed pipeline is shown in **Supplementary Figure 4**. Note as our proposed method utilizes both digital pathology images and molecular information, the resulting pipeline uses two types of DNNs. Finally, cellularity information is shown to improve tumor type and subtype grading performance for the first time in literature.

Accurate classification of LGG grade II from LGG grade III is more challenging as the two tumor types can have a very similar histopathologic appearance. The DNN model used for LGG grade II/III is similar to that of LGG/HGG; however, the network contains more layers which may capture a subtle difference between two similar tumor grades. The network used here with more layers is ResNet at the second step. The detailed structure of these DNNs is listed in **Supplementary Table 1**.

## Dataset

We use 549 whole slide images (WSIs) with molecular information for key alterations (*IDH*, *ATRX*, 1p/19q, and *MGMT* promoter) from The Cancer Genome Atlas (TCGA) dataset in the Genomic Data commons (GDC). The 549 WSIs contain 201 LGG grade II, 229 LGG grade III and 119 HGG grade IV, respectively. We select the top super-pixel as the final ROI of size 1000 × 1000 from WSI following the ROI selection strategy introduced in Methods section. Therefore, we have an overall 549 ROIs for the study. For nuclei segmentation, we utilize UNet architecture (55). The training H&E staining data is obtained from Multi-Organ nuclei segmentation challenge (MoNuSeg), which contains 30 images and around 22000 nuclear boundary annotations for several organ tissues (56). We take one image from the MoNuSeg as a reference, then apply color normalization to the 549 ROI images, so that all objects have a similar color appearance that preserves original structure. The ground truths of the experimental data in this work are obtained from consensus expert ground truths in TCGA. All diagnoses and molecular information are derived directly from the TCGA data set. Diagnoses were made from the contributing institutions and molecular data were obtained using a combination of whole exome sequencing, DNA copy-number analysis, mRNA sequencing, and DNA methylation profiling. A neuropathologist reviewed the histology images and confirmed the validity of the given diagnoses (KJ).

To evaluate the proposed method, we use 5-fold cross validation. The dataset is randomly split into training and testing data based on tumor grade of LGG grade II, LGG grade III, and HGG with ratio 8:2. Moreover, in order to increase data samples, we crop sub-regions of patches with size of 512 × 512. In addition, we also apply data augmentation techniques (random rotation of 90°, 180°, 270°, random flipping image along axis, and random scaling image by 0.95~1.1) to increase the number of training samples. In our experiments, we consider *IDH1/2, ATRX*, 1p/19q, and *MGMT* promoter methylation as the key molecular information. Both *IDH* and *ATRX* has mutant type (MT) and wild-type (WT). The 1p/19 has non-codeletion (NC) and codeletion (CD). The *MGMT* has unmethylated (UM) and methylated (ML) types. The molecular information distribution used in this paper is listed in **Supplementary Table 2**. In the study, there are 154 astrocytomas (AA), 112 oligoastrocytoma (OA), 164 oligodendroglioma (OD), and 119 glioblastomas (GBM), respectively. It is worth noting that oligoastrocytoma is strongly discouraged in new WHO classification (3), but these diagnoses were given at referring institutions prior to 2016. For the purposes of this study, we will be combining astrocytomas and oligodendrogliomas based on grade (e.g. diffuse astrocytoma and oligodendroglioma = lower grade glioma grade II).

## Experiments and Results

All experiments in this study are performed in accordance with relevant guidelines and regulations as approved by the institutional IRB committee at Old Dominion University.

### Nuclei Segmentation and Cellularity

We first apply a UNet to segment nuclei by using the MoNuSeg dataset and then obtain the cellularity feature. **Supplementary Figure 5** shows three cases of nuclei segmentation. The proposed DNN is implemented using PyTorch 1.0 on high-performance cluster with Nvidia V-100 GPU. The minibatch size is set as 2 as the tile size is large and maximum training epoch is set as 80. We use binary cross-entropy as objective function. In training phase, we minimize the cross-entropy loss (57) to optimize the model as follows:

(5) $$loss=-\sum_{\forall x}p\left(x\right)\log\left(q\left(x\right)\right),$$

where p is the true distribution, and q is the estimated distribution of class. In training phase, we use Adam (58) optimizer with initial learning rate of *lr*_0_ = 0.001, and the learning rate (*lr_i_*) is gradually decreased as:

(6) $$lr_{i}=\;lr_{0}*{\left(1-\frac{i}{N}\right)}^{0.9},$$

where *i* is epoch counter, and *N* is a total number of epochs in training.

### Tumor Type Classification

In order to investigate the impact of molecular information to the classification performance, we construct a paired data with/without the genomic information. In addition, we also explore the impact of network by applying a regular CNN and a ResNet for distinguishing HGG *vs*. LGG, and LGG II *vs*. LGG II, respectively. We evaluate the proposed method using five-fold wcross validation. The result summary is shown in **Supplementary Figure 6**.

The performance comparison in **Supplementary Figure 6** shows that ResNet offers better performances than that of regular CNN under the same experimental condition. Fusion of molecular information with pathology consistently improves the classification accuracy. Inclusion of all information (pathology intensity, molecular, and cellularity) achieves the best performance. In comparison, cellularity shows improvement in the ability of ResNet to capture subtle difference among all glioma subtypes and may help to significantly improve the classification accuracy for distinguishing LGG II *vs*. III. The confusion matrix of the proposed method for ResNet with cellularity is shown in **Supplementary Table 3**.

This experiment investigates effect of different combinations of patient data using 5-fold cross validation for tumor type classification. The result is shown in **Supplementary Table 4**. The highest classification accuracies for HGG *vs*. LGG, and LGG II *vs*. LGG III are 93.81% ± 1.98% and 73.95% ± 3.73%, respectively. The small standard deviation indicates robust model performance with minimal overfitting.

### Tumor Subtype Classification

In this experiment, we study the effect of cellularity feature to discriminate between *IDH* mutation status that may indicate glial aggressiveness within a specific type of brain tumor. The results show potential correlation between cellularity and IDH types as shown in **Supplementary Table 5**. It shows the average cellularity value and variance among different grade gliomas. A higher-grade glioma has a higher value of cellularity. For LGG grade III and HGG, cellularity of tumors with wild-type *IDH* is higher that of mutant *IDH*. However, for LGG II, the mutant type *IDH* has a higher value than that of wild type. In recent literature, grade II or III astrocytomas that are *IDH*-wildtype actually show molecular features of glioblastoma and should be considered as glioblastoma despite low cellularity and lack of histologic evidence of malignancy (13).

### Effect of Dilation on Cellularity Computation

In recent literature, morphological dilation step is applied on the malignant nuclei to expand the malignant cancerous cells that may account for the presence of cytoplasm around each nucleus (18, 53). The dilation size is set as 11 as in (18, 53). Cellularity value ranges within 0 and 1. In this study, we also investigate the impact of cellularity with dilation on tumor grading. The average cellularity with different dilation size (0, 10, 12, and 15) is shown in **Supplementary Table 6**. The classification accuracy comparison is listed in the **Supplementary Table 7**. Inclusion of cellularity with dilation size of 12 offers the best performance in both tasks, however, the improvement is trivial comparing to the result without dilation.

The results show the best tumor type classification accuracy is obtained for both LGG *vs* HGG, and LGG grade II *vs* LGG grade III when all different types of patient information in this study (DNN analysis of pathology images, molecular and cellularity) are considered. ResNet offers better classification accuracy for discriminating grade tumor (e.g., LGG grade II and LGG grade III). However, inclusion of cellularity with dilation cannot grant the performance improvement. Choosing a proper dilation size is also challenging. For example, in our experiment, the performances of dilation size of 10 and 15 are smaller than that of without any dilation. According to our experiment and work in (18, 53), the dilation size is recommended as 11 or 12 if needed.

### Molecular Classification

In this section, we investigate molecular classification (IDH status, 1p/19q codeletion, and ATRX status) based on different features extracted from digital pathology images, cellularity, histological type, and tumor grade. We construct a neural network in R with repeated 5-fold classification. The 5-fold cross validation and test results are shown in **Supplementary Tables 8**, **9** respectively.

Our results show that the performance of the molecular classification improves after we add the histology type and the tumor grade information.

### Comparison With State-of-the-Art

We compare our result in this work with existing works in literature as shown in **Supplementary Table 10**. Note the comparison is qualitative rather than quantitative as the patient data, methods, and number of patients are all different for these works. The comparison Table shows that for tumor type classification, our work is comparable in differentiating HGG *vs* LGG, and offers the best performance on distinguishing LGG grade II *vs* LGG grade III. With addition of molecular information, our proposed method offers the highest accuracy for LGG grade II *vs*. grade LGG grade III classification. **Supplementary Table 10** shows that we have the most number of patient cases (549) in this experiment, which is much more than other studies reported in this comparison. The comparison of our work to that of (20) suggest that both use same type (WSI+molecular) information. However, the number of cases in (20) is very small with only 66 cases, and the performance on discriminating HGG *vs* LGG is lower than our work. Furthermore, unlike (20) this current study also include grading of LGG II *vs* LGG III tumor. **Supplementary Table 1** also shows that while (42) offers the best LGG II and LGG III tumor grading, the sample size is small with only 146 patients. Therefore, our proposed method offers competitive performances for both HGG and LGG, and LGG II and LGG grade III classifications using WSI+molecular data, as required by the most recent WHO guidelines, respectively.

## Conclusion

In this work, we propose a DNN-based method for brain tumor classification and grading using both pathology and molecular data following the latest 2016 WHO classification criteria. The classification method, for the first time in literature, integrates a cellularity feature which is derived from morphology of brain tumor histopathology images to improve the performance. We also propose a new ROI selection strategy for histopathology WSIs by utilizing over-segmentation technique. The experiments show that while type of DNN may not be critical in discrimination of low-grade from high-grade glioma, deep learning may have significant impact for discriminating LGG grade II versus LGG grade III tumors. Moreover, it has long been suggested in pathology literature that glioma cellularity increases along with grade, but it has never been proven until now. Even though DNN-based methods outperform the traditional feature-based methods, one of the common concerns is the feature interpretability. The results may be more actionable if the underlying interpretability is also presented to the medical experts. In the future, we plan to develop an interpretable DNN method for glioma subtype classification, and also evaluate the proposed methods using larger patient data to validate the findings in this study for improved tumor classification. Furthermore, the proliferation marker, Ki-67, offers a promising direction in brain tumor grading in recent literature. Integration of the Ki-67 proliferation index for modeling in the current study can be an interesting future work for glioma grading. Finally, we aim to develop an advanced model for CNS tumor classification following the forthcoming WHO brain tumor classification criteria that is expected to follow recommendations of the cIMPACT-NOW soon.

## Data Availability Statement

Publicly available datasets were analyzed in this study. This data can be found here: https://www.tcia.org/.

## Ethics Statement

The studies involving human participants were reviewed and approved by Old Dominion University IRB. The patients/participants provided their written informed consent to participate in this study.

## Author Contributions

LP designed and constructed the experiments and wrote the draft of the manuscript. KJ verified the ground truth of the experimental dataset and revised the manuscript. ZS collected the genomics data from TCGA and reviewed the manuscript. JC reviewed the manuscript. KI designed the experiments, supervised the whole project, and revised the manuscript. All authors contributed to the article and approved the submitted version.

## Funding

This work was partially funded through NIH/NIBIB grant under award number R01EB020683. This work is also partially supported in part by NSF under grants CNS-1828593 and CMMI-1951745, respectively.

## Conflict of Interest

The authors declare that the research was conducted in the absence of any commercial or financial relationships that could be construed as a potential conflict of interest.
