# Supplementary material for: Deep Neural Network Analysis of Pathology Images With Integrated Molecular Data for Enhanced Glioma Classification and Grading
Source: Front Oncol. 2021 Jul 1;11:668694. doi: 10.3389/fonc.2021.668694 (PMC8282424; doi:10.3389/fonc.2021.668694)
Supplement: Supplementary file 1 [file DataSheet_1.docx]

Supplementary Material

# Supplementary Figures and Tables

## Supplementary Figures


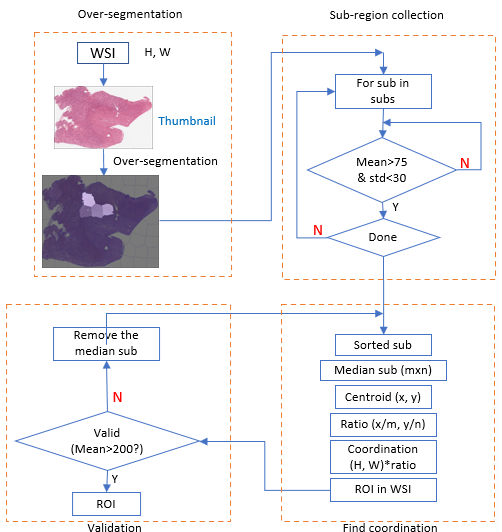


Figure 1. The proposed new strategy for ROI selection from WSI.

a

b


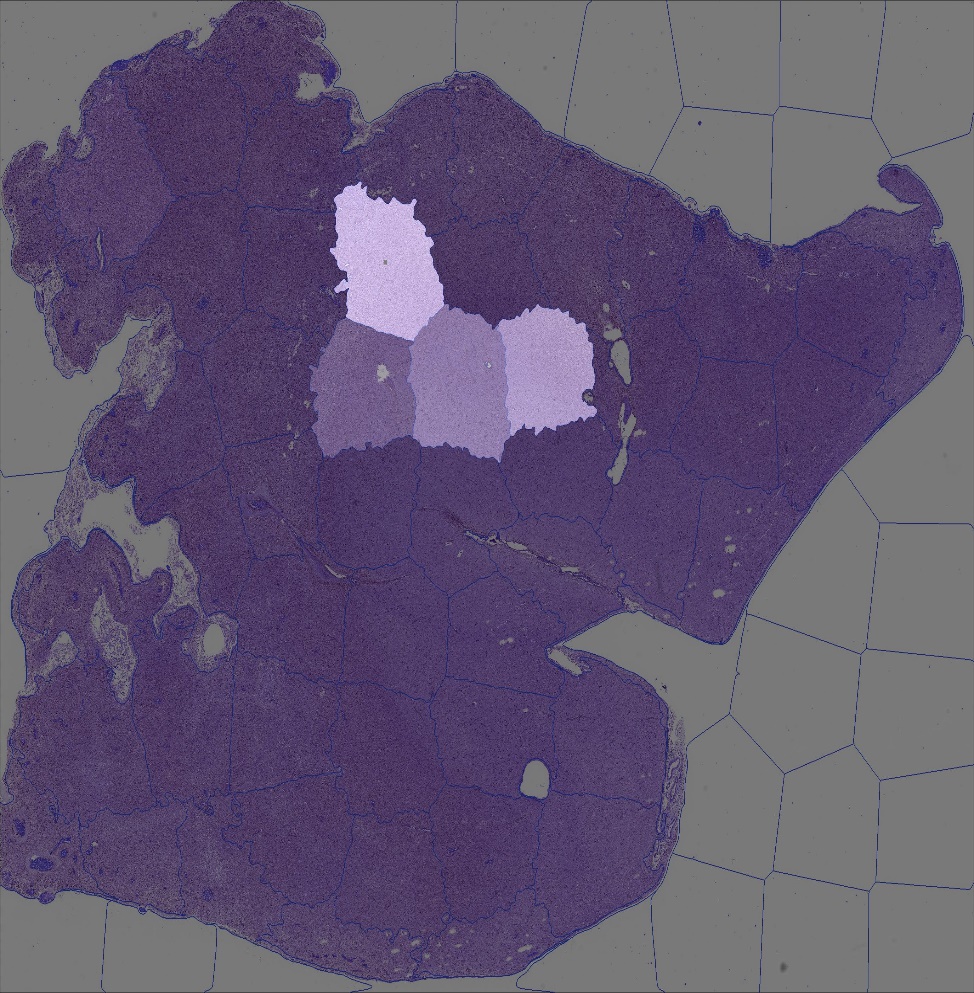

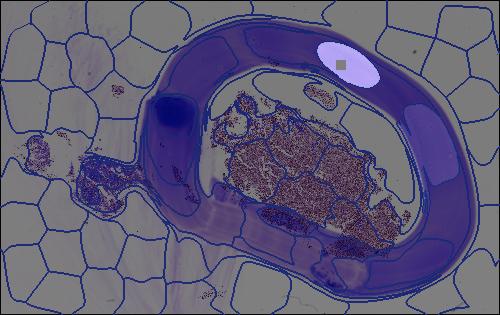


Figure 2. ROI selection using the proposed strategy: (a) a proper ROI selection; and (b) a wrong ROI selection using a pen marker.

Figure 3. Three color normalization instances representing LGG grades II, III, and HGG grade IV. Top row showing original H&E, and bottom row showing the normalized H&E using proposed method: (a) (LGG grade II), (b) (LGG grade III), and (c) (HGG grade IV).


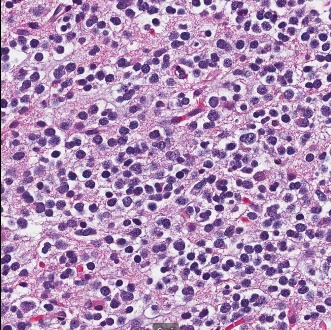

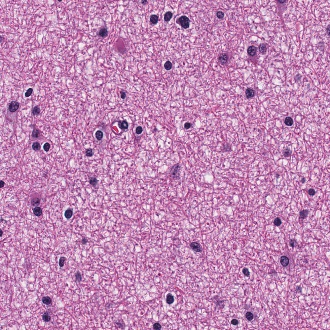

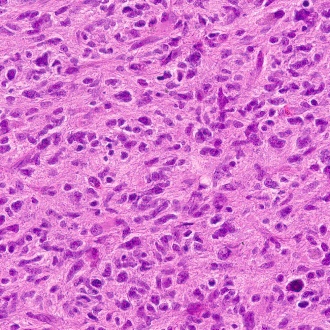

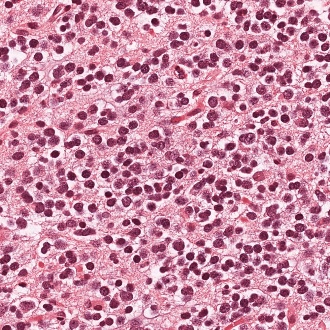

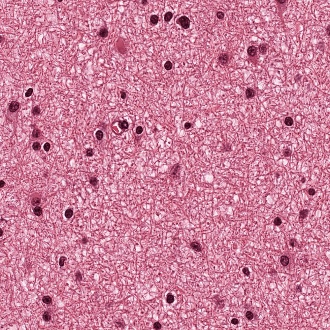

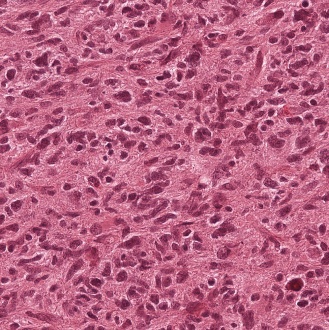


(a) (b) (c)


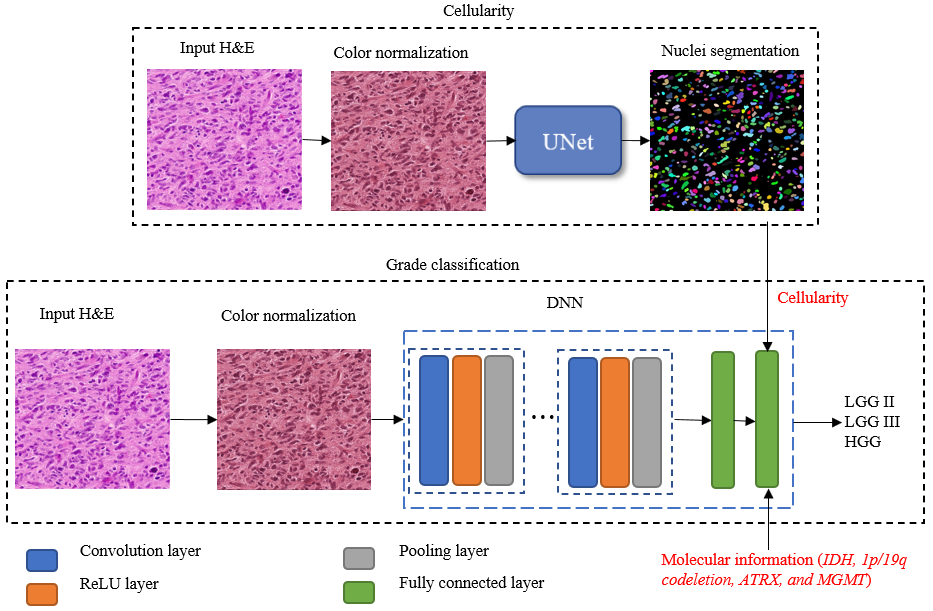


Figure 4. The pipeline has two parts: cellularity and grade classification. In cellularity part, nuclei segmentation of the input H&E is implemented using UNet architecture with Multi-Organ nuclei segmentation data set. For grade classification part, we use a cascaded DNN for distinguish tumor grade. The first DNN (DNN1) is to classify HGG and LGG, and the second DNN (ResNet) is to distinguish LGG II and III. In DNN module, we attach the molecular information (IDH, ATRX, 1p/19q codeletion and MGMT) to the last fully connected layer. For ResNet, there is a residual connection in each block.


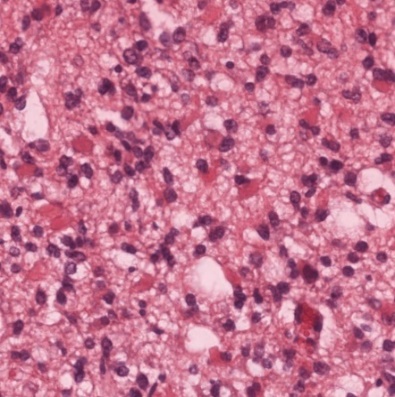

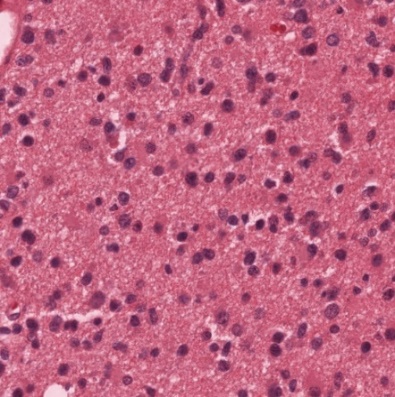

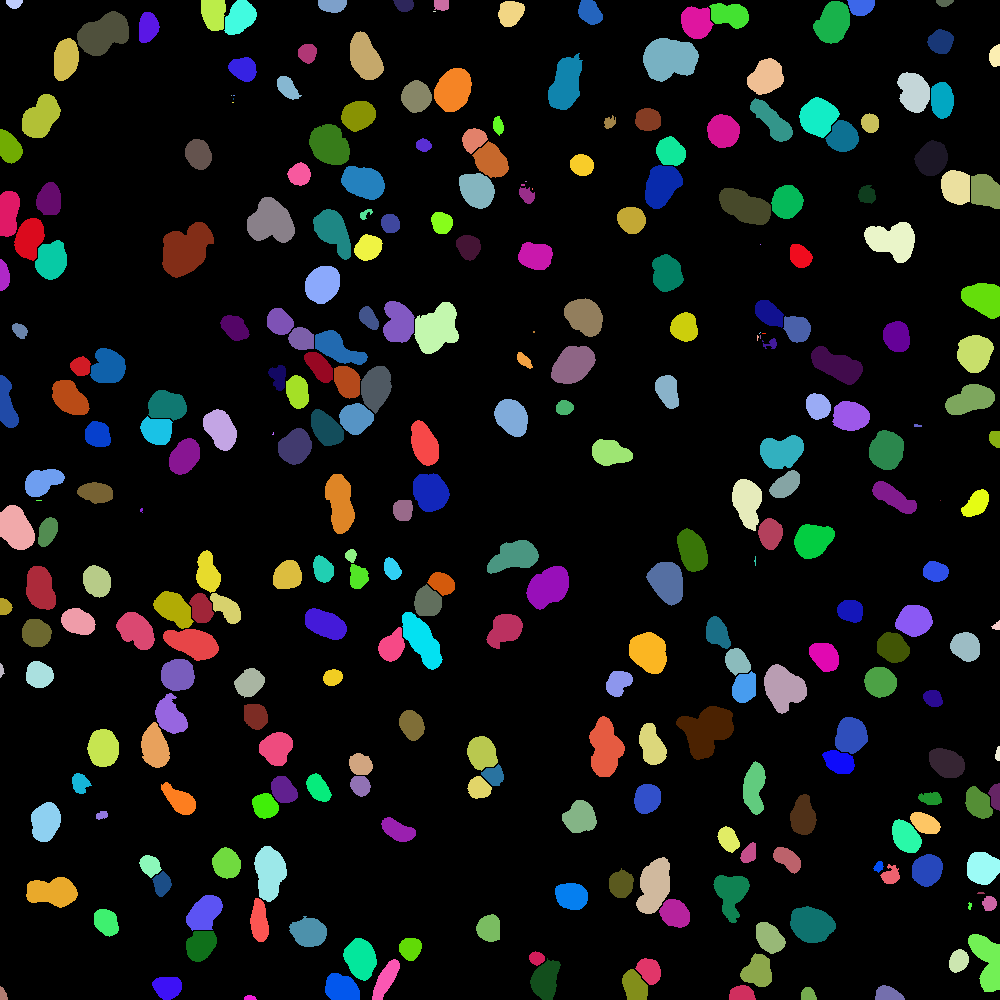

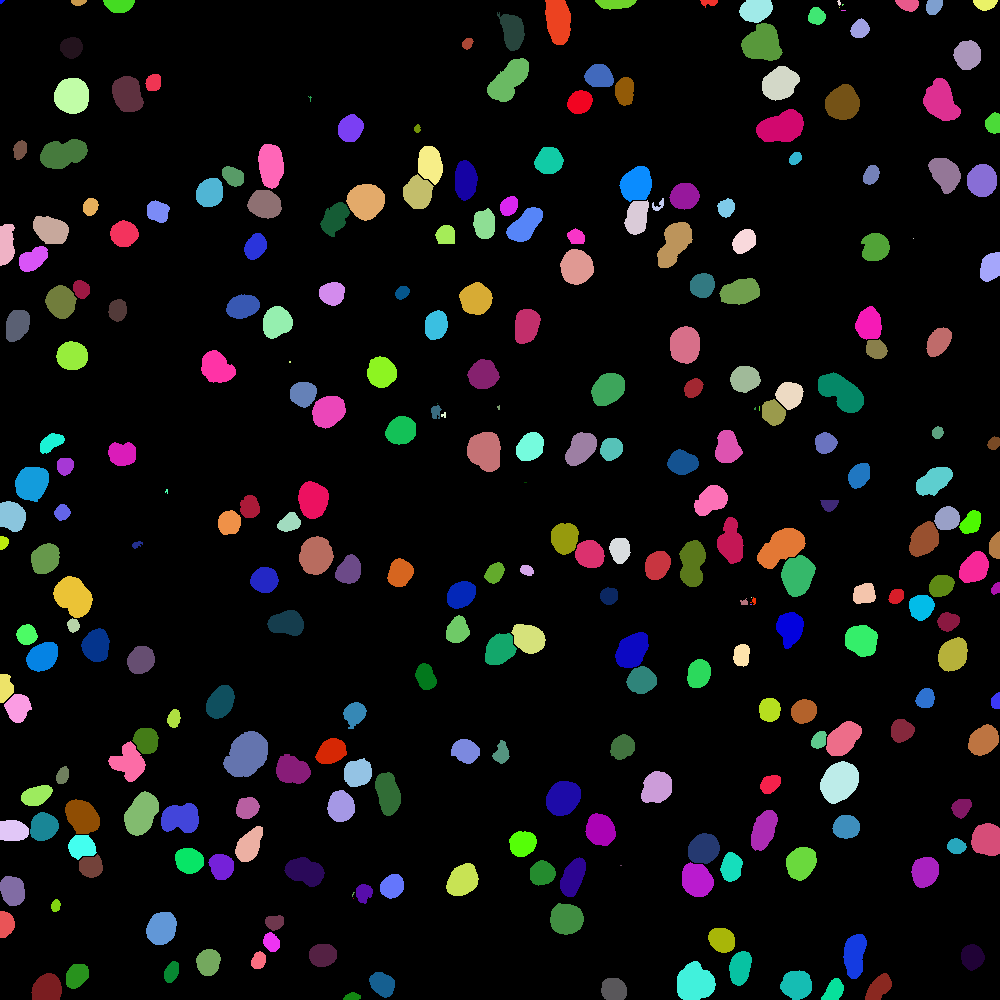

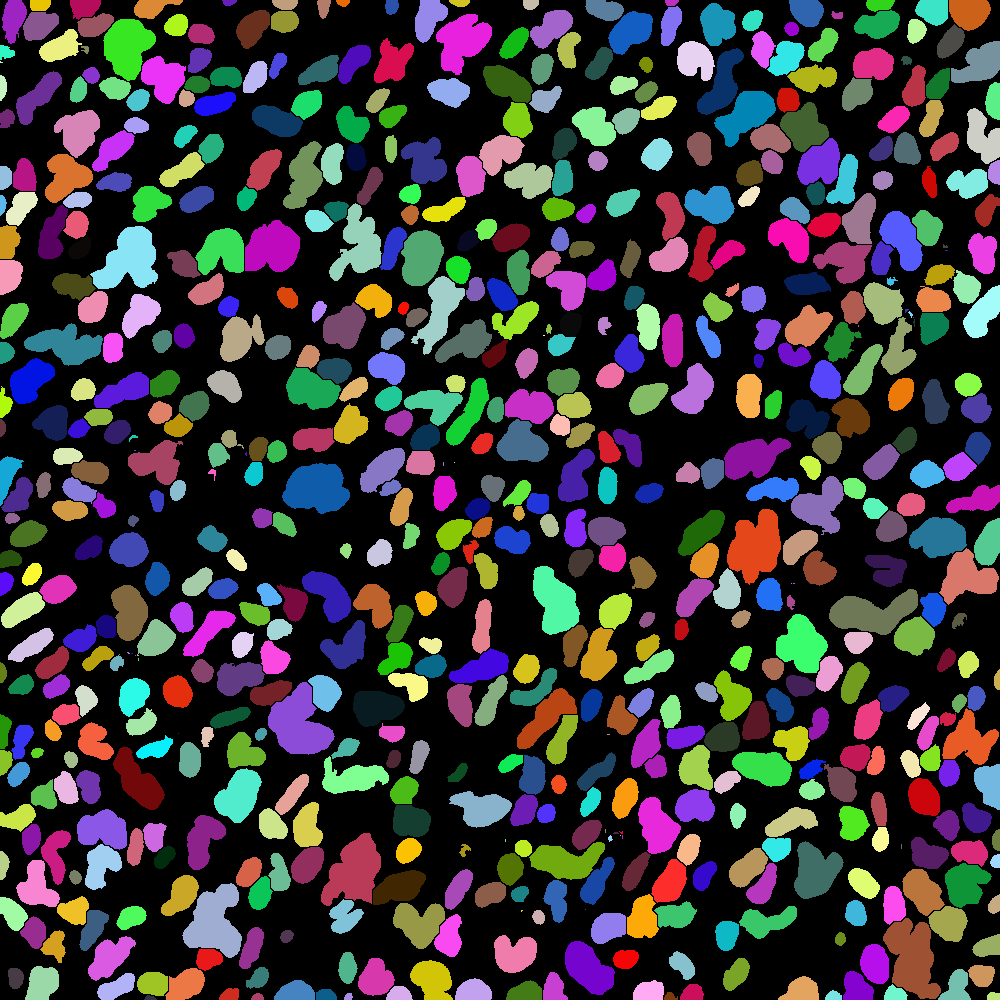

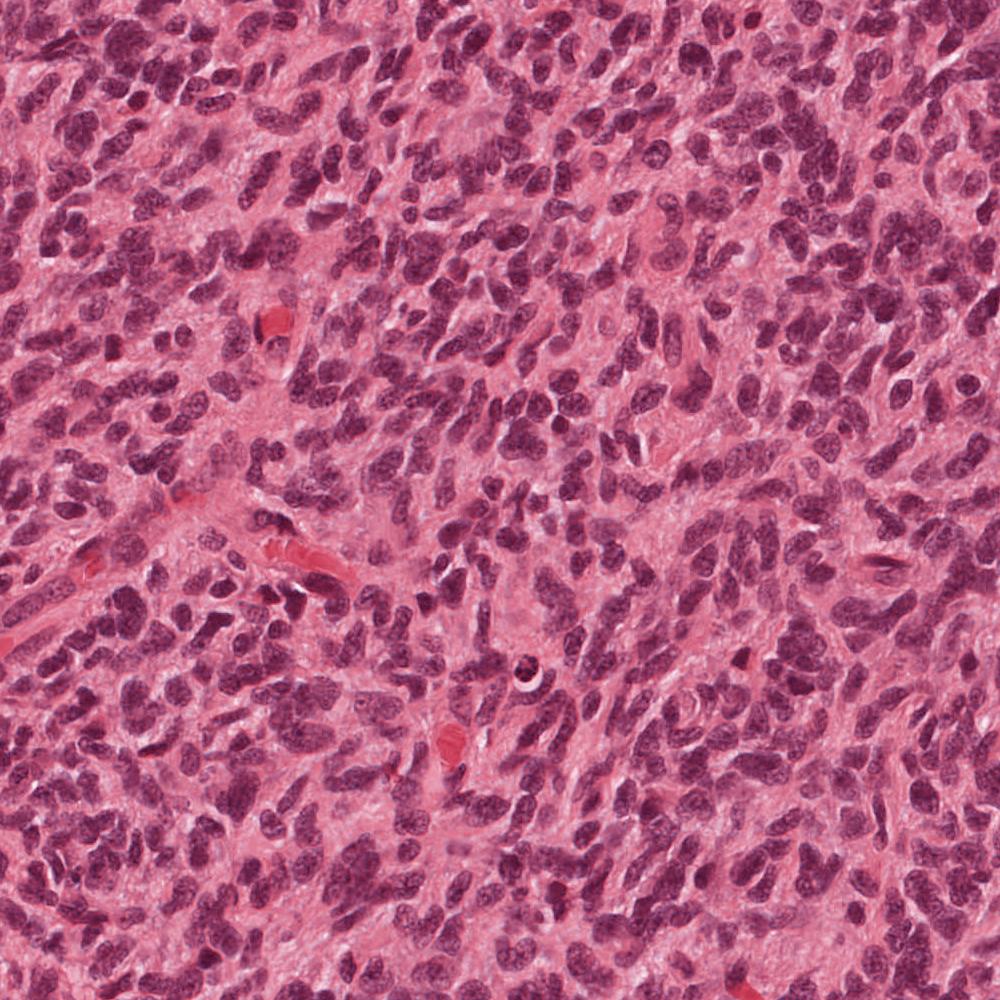


a b c

Figure 5. Three examples of tumor cellularity. Top rows are color normalized image, and bottom rows are corresponding segmented images: (a) LGG II with cellularity of 0.1837, (b) LGG III with cellularity of 0.1441, and (c) HGG with cellularity of 0.4456.


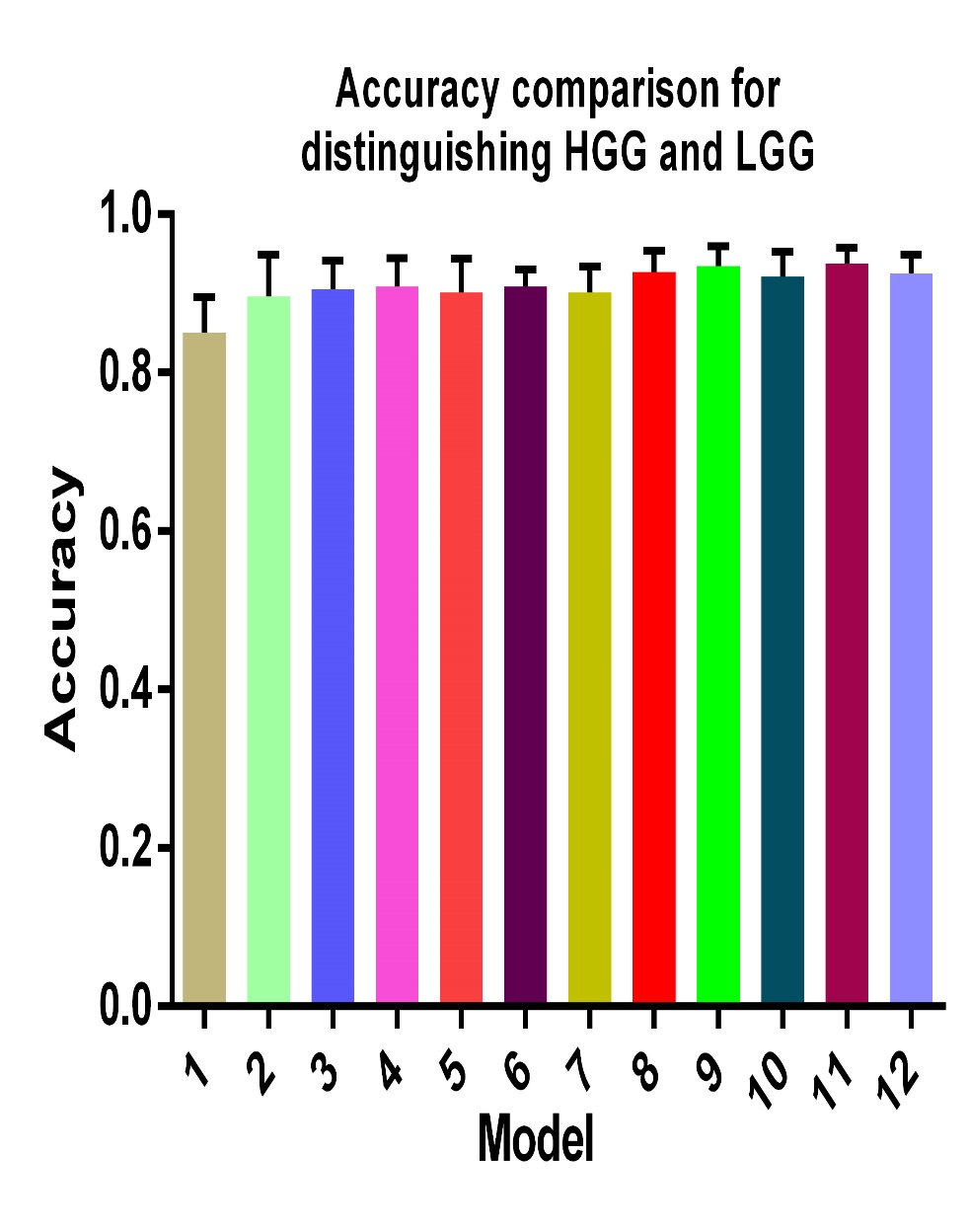

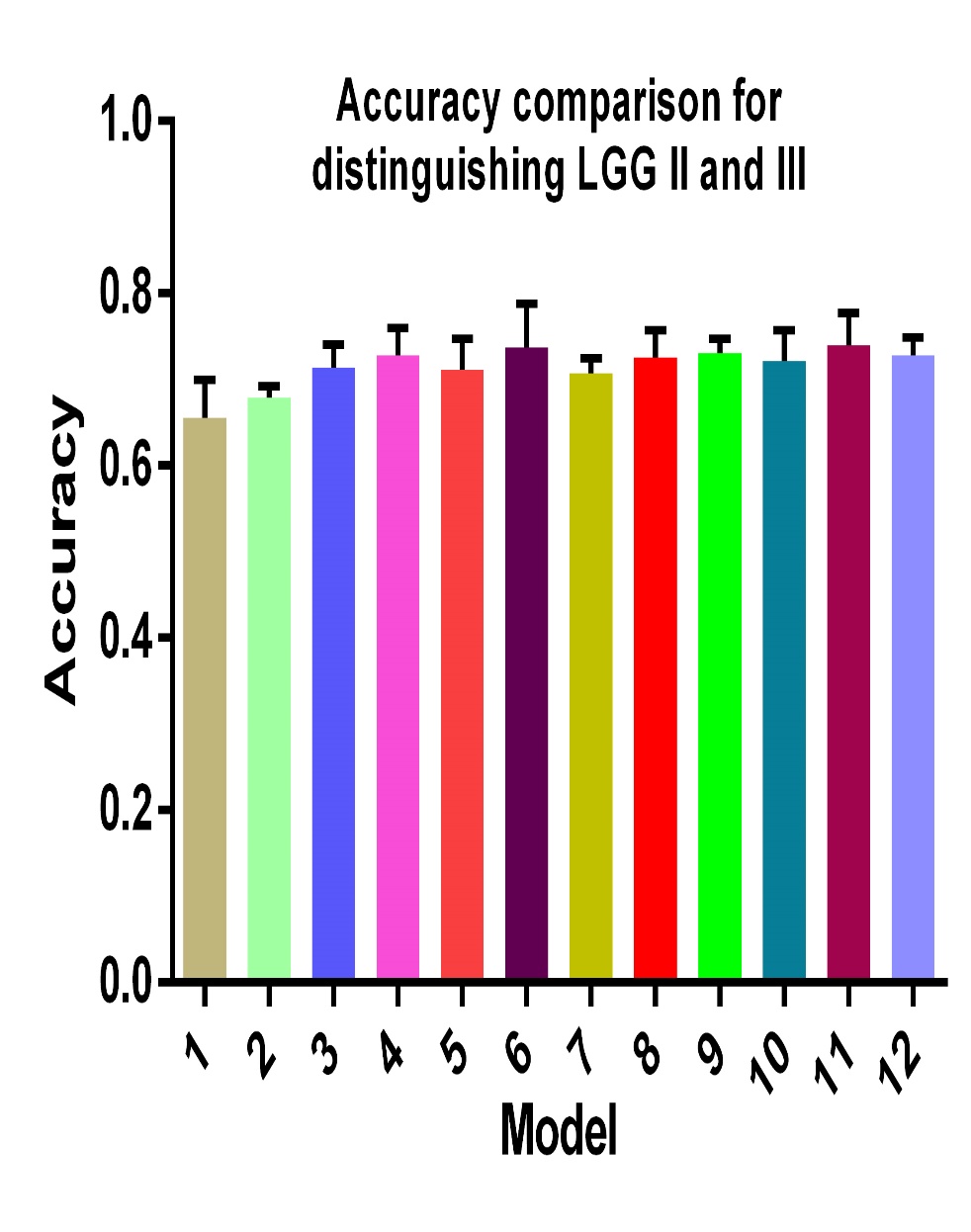


Figure 6. Performance comparison using five-fold cross validation for both HGG vs. LGG (left figure), and LGG II vs. III (right figure). The model 1-6 are regular CNN-based, and the model 7-12 are ResNet-based. Data of model 1 and 7, model 2 and 8, model 3 and 9, model 4 and 10, model 5 and 11, and model 6 and 12 contain pathology, pathology with molecular, pathology with molecular and cellularity, pathology with molecular and cellularity (dilation size as 10), pathology with molecular and cellularity (dilation size as 12), and pathology with molecular and cellularity (dilation size as 15).

## Supplementary Tables

Table 1. Deep neural network structures for CNN and ResNet. We show the data size of each layer. In each convolution block, it has 1 convolution layer, 1 ReLU layer, and 1 max pooling layer. We construct a more complex neural network for differentiating LGG II/III by considering the much similarity within them.

| **Layers** | **Output size of CNN** | **Output size of ResNet** |
| --- | --- | --- |
| Input | $1000\times1000\times3$ | $1000\times1000\times3$ |
| Block 1 | $500\times500\times8$ | $500\times500\times64$ |
| Block 2 | $250\times250\times16$ | $250\times250\times64$ |
| Block 3 | $125\times125\times32$ | $125\times125\times128$ |
| Block 4 | $62\times62\times64$ | $63\times63\times256$ |
| Block 5 | $31\times31\times128$ | $32\times32\times512$ |
| Block 6 | $15\times15\times200$ | $16\times16\times512$ |
| Block 7 | $7\times7\times256$ | $8\times8\times512$ |
| Block 8 | $3\times3\times512$ | $1\times1\times512$ |
| Block 9 | $1\times1\times1024$ | - |
| FC layer 1 | $512$ | 512 |
| FC layer 2 | 16 | $8$ |
| FC layer 3 | 2 | 2 |

Table 2. Molecular data distribution of patient data used in this work.

| Gene | Type | LGG II | LGG III | HGG | Total |
| --- | --- | --- | --- | --- | --- |
| *IDH* | MT | 184 | 163 | 8 | 549 |
|  | WT | 17 | 66 | 111 |  |
| *ATRX* | MT | 85 | 71 | 9 | 549 |
|  | WT | 116 | 158 | 110 |  |
| *1p/19q* | NC | 125 | 162 | 119 | 549 |
|  | CD | 76 | 67 | 0 |  |
| *MGMT* | UM | 28 | 45 | 67 | 549 |
|  | ML | 173 | 184 | 52 |  |

Table 3. Confusion matrix of the proposed method using image patches. In the method, we use CNN for discriminating HGG and LGG, and ResNet for distinguishing LGG grade II and III. Both are with cellularity.

|  | | Actual | | |
| --- | --- | --- | --- | --- |
|  |  | LGG II | LGG III | HGG |
| Predict | LGG II | 149 | 47 | 7 |
|  | LGG III | 45 | 169 | 14 |
|  | HGG | 7 | 13 | 98 |

Table 4. Performance comparison by applying proposed method for different data information. Note: Path. - pathology and cell. - cellularity

| Tumor Type | Data information | Network type | Accuracy |
| --- | --- | --- | --- |
| HGG-LGG | Path. | ResNet | 90.16% ± 3.20% |
|  | Path. + gene | ResNet | 92.72% ± 2.62% |
|  | Path.+ gene+cell. | ResNet | 93.81% ± 1.98% |
| LGG II-LGG III | Path. | ResNet | 70.69% ± 1.72% |
|  | Path. + gene | ResNet | 72.56% ± 3.13% |
|  | Path.+ gene+cell. | ResNet | 73.95% ± 3.73% |

Table 5. Average cellularity and variance for all gliomas with IDH type in our data.

| Tumor  Grade | *IDH* mutant-type  Mutant | *IDH* wild-type  Wild-type |
| --- | --- | --- |
| LGG II | 0.1066±0.0628 | 0.0841±0.046 |
| LGG III | 0.1276±0.0603 | 0.1374±0.0641 |
| HGG | 0.2104±0.1242 | 0.2053±0.095 |

Table 6. Average cellularity with dilation in different size.

| Dilation | LGG II | LGG III | HGG |
| --- | --- | --- | --- |
| 0 | 0.1047 | 0.1304 | 0.2056 |
| 10 | 0.2996 | 0.3684 | 0.5825 |
| 12 | 0.337 | 0.4127 | 0.6351 |
| 15 | 0.4001 | 0.4842 | 0.7099 |

Table 7. Accuracy performance comparison with cellularity in different dilation size. Notation: w-with, g-gene, c-cellularity.

| Task | Dilation size | Information | Average |
| --- | --- | --- | --- |
| HGG vs. LGG | 0 | Res_wg_wc | 0.9344 $\pm$0.0253 |
|  | 10 | Res_wg_wc_10 | 0.9217$\pm$0.0313 |
|  | 12 | Res_wg_wc_12 | 0.9381$\pm$0.0198 |
|  | 15 | Res_wg_wc_15 | 0.9253$\pm$0.0238 |
| LGG II vs. III | 0 | Res_wg_wc | 0.7304$\pm$0.0174 |
|  | 10 | Res_wg_wc_10 | 0.7209$\pm$0.0358 |
|  | 12 | Res_wg_wc_12 | 0.7395$\pm$0.0373 |
|  | 15 | Res_wg_wc_15 | 0.7279$\pm$0.0211 |

Table 8. Five-fold molecular classification validation performance accuracy of the neural network.

| Type of features | IDH Status | 1p/19q codeletion | ATRX Status | MGMT Methylation |
| --- | --- | --- | --- | --- |
| Features of WSI, cellularity, histological type, tumor grade | 84.32 | 84.55 | 72.27 | 78.64 |
| Features of WSI, cellularity | 74.77 | 74.55 | 70.23 | 76.14 |
| WSI | 74.32 | 75.23 | 70.23 | 75.91 |

Table 9. Molecular classification performance accuracy of the neural network for the test dataset.

| Type of features | IDH Status | 1p/19q codeletion | ATRX Status | MGMT Methylation |
| --- | --- | --- | --- | --- |
| Features of WSI, cellularity, histological type, tumor grade | 80.75 | 81.65 | 72.48 | 75.23 |
| Features of WSI, cellularity | 70.64 | 71.56 | 68.81 | 74.31 |
| WSI | 71.56 | 71.56 | 68.81 | 74.31 |

| Authors | Image type | # of patients | Method | Accuracy of HGG vs LGG | Cross-validation | Accuracy of LGG II vs LGG III |
| --- | --- | --- | --- | --- | --- | --- |
| Priya K, *et al.*[11] | MRIs | 231 | SVM | 78.26% | - | - |
| Ertosun, *et al.* [12] | WSI | 7 | CNN | **96%** | - | 71% |
| Basavanhally *et al.* [55] | WSI | 126 | Multifield-of-view classifier | 93% | - | 72% |
| Mousavi, *et al.* [5] | WSI | 138 | Decision-tree | 84.7% | - | - |
| Barker, *et al.* [6] | WSI | 302 | Elastic Net classifier | 93.1% | 5 | - |
| Bagari, *et al.*[56] | MRI+WSI | 20 | CNN | - | - | 90% |
| Reza, *et al.* [22] | MRI+Molecular | 66 | SVM | 86% | 10 | - |
| Reza, *et al.* [16] | WSI+Molecular | 66 | SVM | 93% | 10 | - |
| Wang, *et al.* [41] | WSI+Ki-67 | 146 | SVM | 92.46% | 30 | **96.42%** |
| Our proposed method | WSI+Molecular | **549** | ResNet | 93.81% | 5 | 73.95% |

Table 10. Performance comparison with state-of-art. Accuracy percentage in bold is the best result in the comparison. “-” sign indicates the data is non-available. Bold emphasizes the highest value.
